# Forming a consensus opinion to inform long COVID support mechanisms and interventions: a modified Delphi approach

**DOI:** 10.1016/j.eclinm.2023.102145

**Published:** 2023-08-09

**Authors:** Rebecca Owen, Ruth E.M. Ashton, Francesco V. Ferraro, Lindsay Skipper, Tom Bewick, Paul Leighton, Bethan E. Phillips, Mark A. Faghy

**Affiliations:** a Biomedical and Clinical Science Research Theme, School of Human Sciences, University of Derby, Derby, UK; b Healthy Living for Pandemic Event Protection Network (HL-Pivot), USA; c School of Medicine, University of Nottingham, Nottingham and Derby, UK; d Patient and Public Involvement and Engagement Representative, UK; e Department of Respiratory Medicine, University Hospitals of Derby and Burton NHS Foundation Trust, Uttoxeter Road, Derby DE22 3NE, UK

**Keywords:** COVID-19, Long COVID, Lived experience, Global health, Quality of life, Functional status, Chronic disease

## Abstract

**Background:**

Current approaches to support patients living with post-COVID condition, also known as Long COVID, are highly disparate with limited success in managing or resolving a well-documented and long-standing symptom burden. With approximately 2.1 million people living with the condition in the UK alone and millions more worldwide, there is a desperate need to devise support strategies and interventions for patients.

**Methods:**

A three-round Delphi consensus methodology was distributed internationally using an online survey and was completed by healthcare professionals (including clinicians, physiotherapists, and general practitioners), people with long COVID, and long COVID academic researchers (round 1 n = 273, round 2 n = 186, round 3 n = 138). Across the three rounds, respondents were located predominantly in the United Kingdom (UK), with 17.3–15.2% (round 1, n = 47; round 2 n = 32, round 2 n = 21) of respondents located elsewhere (United States of America (USA), Austria, Malta, United Arab Emirates (UAE), Finland, Norway, Malta, Netherlands, Iceland, Canada, Tunisie, Brazil, Hungary, Greece, France, Austrailia, South Africa, Serbia, and India). Respondents were given ∼5 weeks to complete the survey following enrolment, with round one taking place from 02/15/2022 to 03/28/22, round two; 05/09/2022 to 06/26/2022, and round 3; 07/14/2022 to 08/09/2022. A 5-point Likert scale of agreement was used and the opportunity to include free text responses was provided in the first round.

**Findings:**

Fifty-five statements reached consensus (defined as >80% agree and strongly agree), across the domains of i) long COVID as a condition, ii) current support and care available for long COVID, iii) clinical assessments for long COVID, and iv) support mechanisms and rehabilitation interventions for long COVID, further sub-categorised by consideration, inclusion, and focus. Consensus reached proposes that long COVID requires specialised, comprehensive support mechanisms and that interventions should form a personalised care plan guided by the needs of the patients. Supportive approaches should focus on individual symptoms, including but not limited to fatigue, cognitive dysfunction, and dyspnoea, utilising pacing, fatigue management, and support returning to daily activities. The mental impact of living with long COVID, tolerance to physical activity, emotional distress and well-being, and research of pre-existing conditions with similar symptoms, such as myalgic encephalomyelitis, should also be considered when supporting people with long COVID.

**Interpretation:**

We provide an outline that achieved consensus with stakeholders that could be used to inform the design and implementation of bespoke long COVID support mechanisms.

**Funding:**

None.

**Research in context**

**Evidence before this study**

From our understanding of the area and a search of the literature via PubMed between June 2021 and January 2022, there is a clear lack of effective pharmacological treatments and a sporadic approach to the design and implementation of services for people living with long COVID. A lack of continuity is primarily the result of a lack of pathophysiologic and mechanistic understanding and varied symptom profile that affects patients. With more than 2 million people living with long COVID in the UK and millions more worldwide, there is a need to develop consensus from healthcare professionals, researchers and patients with lived experience in order to develop safe and efficacious support pathways that target improved quality of life.

**Added value of this study**

This Delphi study is the first to provide consensus regarding bespoke support mechanisms and interventions for people living with long COVID. Consensus achieved highlights the importance of specialised care, personalised to the needs of the individual, focussing on individual symptoms. Post-exertional malaise and post-exertional symptom exacerbation should be a key area of consideration, by utilising pacing and fatigue management.

**Implications of all the available evidence**

The consensus offers guidelines that can be incorporated into treatment and support mechanisms to address the long-standing morbidity of long COVID. Our findings can be used alongside potential future pharmacological treatments for long COVID, to improve quality of life and functional status.

## Introduction

The impact of coronavirus disease 2019 (COVID-19) has affected millions of individuals globally and has caused long-standing morbidity in approximately 10% of those with a confirmed diagnosis of COVID-19 within the United Kingdom.^1^, ^2^, ^3^ Post-COVID-condition and/or long COVID, are terms used frequently and interchangeably to describe the continuation or development of new symptoms, 3 months following COVID-19 infection, with symptoms lasting at least 2 months, with no other explanation.^4^ Long COVID, the term first devised by patient groups,^5^, ^6^, ^7^ currently affects ∼2 million people in the UK,^8^ and an estimated ∼150 million globally.^9^ The persistent and episodic symptom profile of long COVID is underpinned by a complex and interacting pathology.^10^^,^^11^ The causes and subsequent impacts of long COVID remain an important area of research to increase the knowledge of proposed mechanisms underpinning pathological changes which include, but are not limited to, organ damage,^12^ endothelial dysfunction,^13^ mitochondrial damage,^14^ formation of microclots,^15^^,^^16^ viral persistence,^17^, ^18^, ^19^ myocardial inflammation,^20^ impaired gas exchange^21^ and immune dysregulation^22^, ^23^, ^24^, ^25^-each of is discussed more detail in a recent review article by Davis et al.^3^

COVID-19 and long COVID affect multiple organ systems, therefore treatment and management pathways will be complex and require input from varying healthcare specialties (general, vascular, respiratory, neurology, immunology).^26^^,^^27^ Due to high demand, there is pressure to develop efficacious support pathways to assist those living with long-standing morbidity caused by long COVID, which will undoubtedly strain healthcare services for many years to come.^8^ Management of long COVID is currently the only approach being offered to patients whilst treatment options are devised.^28^ However, a lack of continuity and guidance remains across healthcare services, despite global efforts being directed at creating multi-disciplinary support pathways.^29^ These issues have led to people living with long COVID reporting self-prescription, turning to a range of over-the-counter medicines, supplements, various therapies, and dietary changes in an attempt to self-manage their symptoms.^30^

To date, a lack of definitive insight and understanding of long COVID pathophysiology and aetiology^30^ propels this to being an emergent threat to global public health.^31^ In light of this urgency, there is a need to determine consensus and consistency in the components of long COVID support pathways to ensure patients receive adequate assistance. Accordingly, this study aimed to establish an expert consensus among medical professionals, people with long COVID, and long COVID academic researchers on the appropriate support mechanisms and potential interventions needed for those living with long COVID.

## Methods

This study was reviewed and approved (ETH2122-0658) by the Human Sciences Research Ethics Committee at the University of Derby. All participants provided written consent in English via the survey platform (JISC Online Surveys) after confirming they understood the study requirements.

### The Delphi process

When there is limited evidence and guidance for a clinical issue, a consensus development technique, such as the Delphi method, can support decision-making and further guidance.^32^ The Delphi process is an acclaimed method to achieve consensus on a clinical issue within healthcare and allows for a flexible approach to gather expert views on a clinical issue.^33^^,^^34^ The process involves repeated communication of statements, which are either accepted or revised/rejected depending on the panel responses until consensus is achieved.^35^ Using an expert-based judgement assumes that the group of experts and varying perspectives will provide a more valid result than from an individual expert.^33^^,^^36^ Consensus guidance allows for standardisation of care, improved outcomes, and facilitation of research.^37^ In this study, practical recommendations generated may be used to assist the organisation of long COVID clinics and optimise the support, management, and treatment of patients.^8^

Modifying the Delphi method is appropriate to ensure the methodology is suitable for the study aims, instead of configuring the study aims to fit the methodology.^38^ The first round of a traditional Delphi typically uses open questioning to identify the focus, however, the present study modified this by the steering group/study management group, including patient and public involvement and engagement (PPIE) reviewing the existing literature, and generating structured statements using a roundtable approach. Free-text boxes were also provided, and experts had the option of commenting on each item in the first round.

PPIE was an integral part of the totality of the design, decision-making, implementation, and analysis of the research process, according to the UK Standards for public involvement. Our PPIE representatives consisted of 4 individuals with lived experience of Long Covid. PPIE involvement included developing and reviewing study materials, obtaining feedback from their networks, assessing terminology, survey length, format, and dissemination of the findings.

### Expert panel selection

The expert panel is defined as a group of individuals with experience or knowledge regarding a particular topic to increase the strength of consensus.^39^ The expert panel criterion included having expertise in COVID-19/long COVID and/or rehabilitation such as academic researchers within this domain, those living with long COVID and healthcare professionals (HCP) (including general practitioners [GP’s], physicians, physiotherapists, and other allied healthcare professionals). Some individuals were allied to more than one of these categories (i.e., a HCP living with long COVID) which formed one distinct expert group. The full breakdown of the expert panel can be found in the results section. The first round of the Delphi study was circulated via social media, word of mouth to long COVID forums, and physician and HCP networks using established links within the research team and project partners. On completion of the first round, participants disclosed which expert they were participating as, and provided an email address to be contacted for subsequent rounds. Round 1 was complete 02/15/2022 to 03/28/22, round two; 05/09/2022 to 06/26/2022, and round 3; 07/14/2022 to 08/09/2022, with experts given ∼5 weeks to complete each round.

### Delphi rounds

The first round consisted of 65 statements over 6 sections (long COVID, long COVID needs, long COVID support, specific rehabilitation interventions for long COVID, long COVID interventions focus, and long COVID rehabilitation inclusion). Using a Likert Scale, experts selected to what degree they agreed with a statement, or how important a statement was. The scale consisted of *‘strongly agree’, ‘agree’, ‘neither agree nor disagree’, ‘disagree’, ‘strongly disagree’, and ‘unsure’*, or for the sections assessing perceived importance *‘very important’, ‘important’, ‘moderately important’, ‘slightly important’, ‘not at all important’, ‘neither’*, and *‘unsure’*. Anonymised results were downloaded from JISC and reviewed by the research team. Items with a response greater than 80% for *‘strongly agree’* and *‘agree’* were taken as achieving consensus, with all other items revised and recirculated following analysis of the open text responses. Open-text responses were not analysed using a formal process but were considered by the trial steering group using a roundtable approach.

For rounds two and three, following analysis of the open text responses within round one, five key terms were defined (rehabilitation, myalgic encephalomyelitis/chronic fatigue syndrome (ME/CFS), post-exertional malaise (PEM), post-exertional symptom exacerbation (PESE), graded exercise therapy (GET)). Additionally, the option of *‘unsure’* and *‘neither’* were removed from the Likert scale. The use of the term ‘Rehabilitation Interventions’ was adapted to ‘Support Mechanisms and Rehabilitation Interventions’.

The survey link with revised statements was sent to the previous round of respondents.

### Role of the funding source

There was no funding source for this study. RO, MF, BP, and RA had access to the dataset and had the final responsibility for the decision to submit for publication. Standards for reporting qualitative research (SRQR) guidelines were adhered to, as well as Delphi specific guidelines available within the literature.^32^, ^33^, ^34^^,^^36^^,^^39^^,^^40^

## Results

### Summary of rounds

Table 1 shows the summary of responses for each round, including the round aim, the number of statements, the number of statements that reached consensus, the number of statements modified for the subsequent round, and the number of statements removed or rejected from that round. In round one, 33 statements were accepted, with 32 revised by the research group using the qualitative free-text responses, and modified for round two. In round two, 17 statements were accepted with 15 statements modified. In round three, 5 statements were accepted and 10 rejected.

**Table 1:** Summary of responses for each round.

	Number of statements	Round aim	Statements that reached consensus (<80%)	Statements modified for the next round	Statements removed or rejected
Round 1	65	Exploratory	33	32	0
Round 2	32	Clarifying	17	15	0
Round 3	15	Clarifying and confirmatory	5	0	10

### Response rate

Overall, there were 273 responses to round one, 186 responses to round two (31% attrition from round 1), and 138 responses to round three (25% attrition from round 2). Across the three rounds, the expert panel consisted of 60–62% of people with long COVID (round 1, n = 164; round 2, n = 115; round 2, n = 83) [PwLC], 12–16% Health Care Professionals living with long COVID [HCP/PwLC] (round 1, n = 33, round 2, n = 30, round 3, n = 25), 15–21% Health Care Professionals [HCPs] (Physiotherapists [5–8%], Physicians [4–7%], GPs [1%], other HCP [5%], round 1, n = 55; round 2, n = 26; round 3, 20) and 7–8% long COVID Researchers [A/R] (round 1, n = 21, round 2, n = 15, round 3, n = 10). Throughout the three rounds, participants represented every region within England, with the majority residing in South England (26.4–29.7% round 1, n = 72, round 2, n = 53, round 3, n = 41), followed by the Midlands (24.2–28.2% round 1, n = 77; round 2, n = 45; round 3, n = 36), North England (13.4–14.3%, round 1, n = 39; round 2, n = 25; round 3, n = 19) and East of England (4–4.8% round 1, n = 11; round 2, n = 9, round 3, n = 6). Participants also represented Scotland (7.7–10.2% round 1, n = 21, round 2, n = 19, round 3, n = 13) and Wales (0.5–1.8%, round 1, n = 5, round 2, n = 1; round 3, n = 2), and a further 15.2–17.2% (round 1, n = 47, round 2, n = 32; round 3, n = 21) of participants resided outside of the UK (United States of America [USA], Austria, Malta, United Arab Emirates [UAE], Finland, Norway, Malta, Netherlands, Iceland, Canada, Tunisie, Brazil, Hungary, Greece, France, Australia, South Africa, Serbia, and India).

### Summary of results

Consensus was reached on 55 statements overall. For ease of understanding and comprehension, statements were merged where relevant to form a final list of 44 as displayed in Table 2. These statements can be considered in four domains: i) long COVID as a condition (n = 6), ii) current support and care available for long COVID (n = 3), iii) clinical assessments for long COVID (n = 3), and iv) support mechanisms and rehabilitation interventions for long COVID (n = 13), with 19 further statements related to iv) divided into three sub-domains: a) what these should consider (n = 4), b) include (n = 9), and c) focus on (n = 6). Full response breakdown including % agreement, and when consensus was achieved for each round is available in Supplementary Tables S1–S6.

**Table 2:** Accepted statements across the three rounds.

Domain	Statement
Long COVID as a condition	- Long COVID is a public health concern.
	- Long COVID is a condition that will require support for patients’ long term (6+ months).
	- Long COVID is a condition that affects multiple systems of the body, presenting itself through several symptoms.
	- Long COVID is a condition that affects individuals of good health prior to contracting COVID-19. - Long COVID cannot be predicted by the severity of symptoms during the acute phase (first 2 weeks) of COVID-19 infection.
	- It is unknown whether individuals living with long COVID will make a full recovery.
Current support and care available	- There is inadequate and inconsistent support amongst all healthcare services for individuals living with long COVID.
	- There is a lack of clear referral pathways to support people living with long COVID throughout all healthcare settings.
	- There is a lack of understanding from healthcare professionals on how to support people with long COVID.
Clinical assessment for long COVID	- People living with long COVID require detailed clinical assessments and functional screening assessments which should be considered during diagnosis and treatment.
	- Respiratory function should be assessed to establish rehabilitation needs for people living with long COVID.
	- People with long COVID should complete a formal assessment of physical and emotional functioning to identify rehabilitation needs.
Support mechanisms and rehabilitation interventions for long COVID	- Long COVID requires specialised and comprehensive rehabilitation interventions, that should be guided by the needs of the patient and created with patient input.
	- Long COVID rehabilitation and support mechanisms should be dependent on each individuals’ symptoms.
	- Long COVID support and rehabilitation should be individualised to the patient’s needs.
	- Those completing long COVID rehabilitation and support interventions should have regular communication and monitoring with care providers.
	- Long COVID services should offer psychological well-being support for patients who require it.
	- People living with long COVID should receive adequate support from their GP.^a^
	- Long COVID support should adopt a multidisciplinary approach (e.g., including physiotherapists, clinicians, rehabilitation specialists and exercise scientists working together).
	- Long COVID rehabilitation intervention should be personalised according to age and comorbidities (i.e., pre-existing medical conditions).
	- Those undergoing long COVID rehabilitation should be closely monitored to establish whether their condition is improving, deteriorating or neither.
	- Long COVID rehabilitation might be different for each individual.
	- Improving quality of life and physical function is a key aim of long COVID rehabilitation.
	- Patients in hospital with COVID-19 should receive tailored rehabilitation and support before being discharged.
	- Individuals experiencing symptoms consistent with ME/CFS^b^ and PEM^c^ should be carefully supported before participating in physical activity.
Long COVID rehabilitation should focus on:	- Breathlessness
	- Cognitive dysfunction (thinking, remembering, learning, attention confusion)
	- Fatigue
	- Respiratory function
	- Restoring functional capacity
	- Sleep disturbance
Long COVID rehabilitation and support mechanisms should include:	- Advice on modifying/adapting daily activities such as using aids to allow greater functional ability. - Self-management of daily living
	- Cognitive (regulating energy use for activities that involve mental capacity e.g., thinking, understanding, learning, remembering) and physical (regulating energy use for physical activity or tasks) pacing of activities.
	- Support returning to work
	- Support returning to normal activities of daily living
	- Breathing techniques and relaxation techniques (meditation, mindfulness)
	- Fatigue management
	- Patient preference on how they attend their interventions and support, and what is most suitable for them at the time.
	- A model that contains face to face and virtual sessions.
Long COVID rehabilitation and support mechanisms should consider:	- The mental impact of living with long COVID
	- Tolerance to physical activity
	- Emotional distress and wellbeing
	- Research of pre-existing conditions with similar symptoms e.g., ME/CFS^b^

a General practitioner.

b Myalgic Encephalomyelitis/Chronic Fatigue Syndrome).

c Post exertional malaise.

The 10 statements that did not reach consensus by the end of round 3, and as such were rejected, are presented in Table 3.

**Table 3:** Rejected statements (<80% agreement).

Domain	Statement
Long COVID as a condition	- If regular physical activities do not provoke symptoms or post exertional symptom exacerbation, then people with long COVID can participate in their regular physical activities.
Support mechanisms and rehabilitation interventions for Long COVID	- Those designing support mechanisms for long COVID can learn lessons from other acute respiratory infections (e.g., pneumonia).
	- Those designing support mechanisms for long COVID can learn lessons from other chronic respiratory diseases (e.g., asthma and chronic obstructive pulmonary disease).
Long COVID rehabilitation and support mechanisms should include:	- Low level physical activities (e.g., walking) that result in moderate increases in heart rate.
	- Activities incorporating muscle use.
	- Support to increase flexibility and functional movement proficiency.
	- Advice on nutrition and diet to support recovery.
	- Interventions should be delivered face to face and make use of specialist facilities and personnel.
	- Interventions that can be completed remotely and away from clinical settings.

### Between group discrepancies

Across the three rounds, 11 statements reached overall consensus using the established criteria but this was not universal across each expert group. Each statement was considered individually to determine the extent to which expert groups did not reach consensus, and for transparency the data is included in Table 4. Furthermore, when international responses (15–17% across rounds) were excluded from the dataset, one statement did not reach overall consensus in round three: ‘Long COVID support mechanisms and rehabilitation interventions should include a model that contains face-to-face and virtual sessions’.

**Table 4:** Accepted statements reaching overall concensus (>80%) with discrepancies between groups.

Round 1 (reached overall consensus)	Overall	HCPs^a^ N = 55	PwLC^b^ N = 164	HCP/PwLC^c^ N = 33	A/Rs^d^ n = 21
Long COVID is an illness that requires specialised rehabilitation interventions.	86%	84%	89%	91%	**62%**
Respiratory function should be assessed to establish rehabilitation needs for people living with long COVID.	81%	**69.1%**	84%	91%	**71%**
People living with long COVID should complete a formal assessment of physical and emotional functioning to identify rehabilitation needs.	88%	91%	88%	91%	**72%**
People living with long COVID should receive adequate support from their GP.^e^	87%	81.8%	90%	91%	**71%**
People living with long COVID should receive a comprehensive rehabilitation programme.	88%	85.1%	91%	85%	**72%**
How important is it for long COVID rehab to focus on respiratory function	86%	**75%**	87%	85%	85%
How important is it for long COVID rehab to include breathing techniques	85%	**78%**	87%	91%	91%
**Round 2 (reached overall consensus)**	**Overall**	**N** = **55**	**N** = **164**	**N** = **33**	**N** = **21**
There is a lack of clear referral pathways to support people living with long COVID throughout all healthcare settings.	87%	**69%**	85%	100%	100%
How important is it for long COVID support and rehabilitation interventions to include relaxation techniques and breathing techniques (e.g., meditation, mindfulness)	81%	81%	85%	**77%**	**60%**
**Round 3 (reached overall consensus)**	**Overall**	**N** = **20**	**N** = **83**	**N** = **25**	**N** = **10**
Long COVID cannot be predicted by the severity of symptoms during the acute phase (first 2 weeks) of COVID-19 infection.	88%	**75%**	90%	96%	80%
How important is it for long COVID support and rehabilitation interventions to include a model that contains both face to face and virtual sessions	80%	85%	**79%**	84%	**70%**

Consensus not reached within group (<80%) highlighted Bold.

a Healthcare Professionals.

b People living with long COVID.

c Healthcare Professionals living with Long COVID.

d Academics/Researchers working within long COVID.

e General Practitioner.

## Discussion

The present study used a modified Delphi method to obtain consensus opinion for the development and refinement of long COVID support pathways within the UK. Fifty-five statements related to long COVID reached consensus by a panel of experts in the domains of long COVID as a condition, care and support available for long COVID, clinical assessment for long COVID, and support mechanisms and rehabilitation interventions for long COVID. Whilst research regarding long COVID as a condition is available,^7^ there is an absence of current pharmacological interventions for long COVID, therefore the novelty of this Delphi study has potential clinical value in the development of support pathways that are consistent, safe, and efficacious to service users.

The consensus reached in this study agrees with existing literature that it is likely that long COVID will have a substantial impact on public health.^31^^,^^41^ Also consistent with other research, the expert panel agreed that long COVID is a condition that affects multiple systems of the body, presenting itself through several symptoms, and that those living with long COVID will require long-term support.^3^ The panel also agreed that it is unknown whether individuals will make a full recovery.^3^ When diagnosing and treating long COVID, our consensus statements suggest that a detailed clinical assessment, medical investigations, laboratory testing, and functional screening should be undertaken to provide an outline of the individual patient needs. These must include formal assessments of respiratory function, functional status, and emotional state. This compliments the work of Davis and colleagues^3^ who highlight the important need for further research that builds on the existing knowledge of the appropriate tests for long COVID. In this work, the authors highlight that this should include detailed neuroimaging, metabolic profiling, and nanoneedle diagnostic testing. Furthermore, both studies highlight that long COVID support pathways are inconsistent across healthcare settings, and lack a clear referral/re-referral pathway and understanding from healthcare professionals on how to support those living with long COVID. The outcomes of the study presented herein provide detail that can be incorporated into treatment and management guidelines to address broad issues and improve quality of life for people with long COVID within the UK.

A further finding of this study is the consideration of symptoms of ME/CFS and PEM, also present in long COVID.^42^ The panel agreed that individuals experiencing symptoms consistent with ME/CFS and/or PEM should be thoroughly examined and monitored before being encouraged to participate in physical activities or exercise. However, the panel did not reach consensus that when regular physical activities do not provoke symptoms of PESE, then those with long COVID can participate in their regular physical activities and was therefore rejected in round three. Similarly, in line with existing research that exercise may be detrimental for people with long COVID and ME/CFS, or PEM^43^^,^^44^ the panel disagreed that long COVID support mechanisms should include low-level physical activities that result in moderate increases in heart rate, activities incorporating muscle use, and support to increase flexibility and functional movement proficiency, but plans should be individualised and tailored to the needs of the patient. PESE and PEM are commonly experienced by those with long COVID^44^ and presents a significant challenge such as reduced capacity to work, and reduced physical and social functioning.^45^ Furthermore, experts agreed that there should be consideration of the research of pre-existing conditions with similar symptoms such as ME/CFS, but not respiratory conditions such as COPD, asthma, and pneumonia.

According to this study, support mechanisms and interventions should be personalised for each patient, and include detailed specialist input. Where appropriate, interventions should include advice on modifying/adapting daily activities such as using aids to allow greater functional ability, self-management of daily living such as support returning to work and normal activities of daily living, cognitive and physical pacing of activities, fatigue management, and breathing and relaxation techniques. Experts agreed that breathlessness, cognitive dysfunction, fatigue, respiratory function, restoring functional capacity, and sleep disturbance should be some of the focuses of the support and interventions, with psychological well-being support available for those who require it. This recommendation is in line with the existing literature suggesting that exhaustion, cognitive dysfunction, chest pressure and/or tightness, and dyspnoea are the most common symptoms of long COVID.^46^ Additionally, the mental impact of living with long COVID, tolerance to physical activity and emotional distress and well-being should be considered as part of a holistic and interdisciplinary support pathway.

This Delphi study concludes that long COVID support should adopt an interdisciplinary approach that brings together clinicians, healthcare practitioners, and rehabilitation experts including clinical exercise specialists, as well as the patients receiving informed support from their GP. Adopting an interdisciplinary approach is beneficial in supporting an already strained national health service^47^ as collaborative approaches can extend knowledge, and best utilise space and facilities to conduct detailed and integrative assessments.^47^, ^48^, ^49^ Support mechanisms should be delivered via a model that contains face-to-face and virtual sessions, with patient preference on how they attend their interventions and support. When completing interventions for long COVID, patients should have regular communication with health care professionals, with adequate monitoring to establish impact.

The Delphi method is a flexible approach with anonymity being a key feature of the method. However, due to the need to identify respondents and non-respondents for participation in consecutive rounds, quasi-anonymity was used in the present study.^40^ Therefore within the current study, respondents were known to the researcher, but their judgements and responses remained anonymous. Additionally, majority of respondents and the author list reside in a high-income country (UK), therefore this does not reflect complete global utility and subsequent investigation would be required for service development and implementation more broadly.

One consideration for the current study is that 11 statements that reached overall consensus did not reach consensus within every group as shown in Table 4. Specifically, 7 of these statements did not reach consensus amongst the academics and researchers, potentially explained by the smaller sample size (n = 21) compared to other groups. Additionally, 5 of these statements did not achieve consensus by HCPs, 1 by PwLC and 1 by HCP/PwLC. These statements should be considered with caution.

In conclusion, this study has achieved consensus regarding the appropriate support mechanisms and rehabilitation interventions for long COVID. The outcomes of this study provide detail that could be incorporated into treatment and management guidelines to address long-standing issues and improve quality of life for people with long COVID within the UK.

## Contributors

RO, MF, BP, and RA completed statistical analysis and revisions, with access and verification of all underlying data. RO wrote the first draft of the report, with input from MF, BP, RA, and LS. RO, MF, BP, and RA developed the concept of the study, with PL guiding the Delphi process. RO, MF, BP, RA, TB, LS, and FF completed a review of the literature and generated the first round statement using a round table approach. Authors were involved in the circulation of the Delphi, using links to external networks (TB to physician and HCP networks, LS to patient and physiotherapist networks, RO, MF, RA, BP, and FF to patient and researcher networks).

## Data sharing statement

Data will be available with publication, with investigator support with a signed data access agreement.

## Declaration of interests

All authors declare no competing interests.
