# Supplementary material for: Forming a consensus opinion to inform long COVID support mechanisms and interventions: a modified Delphi approach
Source: eClinicalMedicine. 2023 Aug 9;62:102145. doi: 10.1016/j.eclinm.2023.102145 (PMC10432807; doi:10.1016/j.eclinm.2023.102145)
Supplement: Supplementary Tables S1–S6 [file mmc1.docx]

**Supplementary tables**

**Table S1:** **Percentage agreement for statements that achieved consensus (>80%) following Round 1 (N=33).**

|  | **Agree** | **Neither** | **Disagree** | **Unsure** |
| --- | --- | --- | --- | --- |
| Long COVID is a condition that will require support for patients’ long term (6+ months). | 96.7% | 2.6% | 0.7% | 0.7% |
| Long COVID is a public health concern. | 96.6% | 0.7% | 1.8% | 0.7% |
| Long COVID is a condition that affects multiple systems of the body. | 99.3% | 0.0% | 0.8% | 0.4% |
| Long COVID is a condition that affects individuals of good health prior to contracting COVID-19. | 91.6% | 5.1% | 2.5% | 1.1% |
| Long COVID is an illness that presents itself through several symptoms. | 98.2% | 1.1% | 1.1% | 0.4% |
| Long COVID is a condition that requires specialised rehabilitation interventions. | 86.1% | 5.90% | 3% | 4.40% |
| People living with long COVID require clinical assessments and medical investigations. | 95.2% | 2.6% | 0.8% | 1.5% |
| Respiratory function should be assessed to establish rehabilitation needs for people living with long COVID. | 81% | 7.7% | 7.4% | 4% |
| People living with long COVID should complete a formal assessment of physical and emotional functioning to identify rehabilitation needs. | 88% | 6.2% | 4% | 1.8% |
| Long COVID intervention should be implemented as early as possible. | 92.3% | 3.7% | 1.9% | 2.2% |
| People living with long COVID should receive adequate support from their GP. | 87.2% | 3.3% | 7.7% | 1.8% |
| People living with long COVID should receive a comprehensive rehabilitation programme. | 87.5% | 5.5% | 3.3% | 3.7% |
| Long COVID support should adopt A multidisciplinary approach. | 94.1% | 1.8% | 2.6% | 1.5% |
| Long COVID rehabilitation plans should be made with patient input. | 98.5% | 0.0% | 1.5% | 0.0% |
| Early implementation of rehabilitation interventions should be encouraged for those with long COVID. | 87.6% | 2.6% | 4.8% | 5.1% |
| Long COVID rehabilitation intervention should be personalised according to age and comorbidities. | 91.1% | 2.6% | 1.4% | 4.8% |
| Those undergoing long COVID rehabilitation should be closely monitored to establish whether their condition is improving deteriorating or neither. | 93.4% | 3.3% | 1.6% | 0.7% |
| Long COVID rehabilitation might be different for each patient. | 98.40% | 1.1% | 1.1% | 0.4% |
| Improving quality of life and physical function is a key aim of long COVID rehabilitation. | 94.60% | 3.3% | 1.5% | 0.7% |
| Long COVID rehabilitation interventions should be guided by the needs of the patient. | 97.1% | 1.1% | 1.1% | 0.7% |
| **How important is it for long COVID rehabilitation to focus on the following…** | **Important** | **Neither** | **Not Important** | **Unsure** |
| Breathlessness | 97.2% | 1.9% | 0.0% | 1.5% |
| Cognitive dysfunction | 96.4% | 1.8% | 0.4% | 1.5% |
| Fatigue | 97.4% | 1.8% | 0.0% | 0.7% |
| Respiratory function | 96.3% | 1.8% | 0.7% | 1.1% |
| Restoring functional capacity | 97.2% | 1.5% | 0% | 1.5% |
| Sleep disturbance | 96.7% | 1.5% | 0.7% | 1.1% |
| **How important is it for long COVID rehabilitation to include the following…** | **Important** | **Neither** | **Not Important** | **Unsure** |
| Self-management of daily living | 96.3% | 1.1% | 0.4% | 2.2% |
| Cognitive pacing | 96.3% | 0.7% | 0% | 3% |
| Physical pacing of activities | 95.6% | 1.5% | 0.7% | 2.2% |
| Support returning to work | 96.3% | 1.1% | 0.4% | 2.2% |
| Support returning to normal activities of daily living | 96.4.% | 1.1% | 0.7% | 1.8% |
| Breathing techniques | 96.5% | 1.1% | 0.4% | 2.9% |
| Fatigue management | 97.4% | 1.1% | 0.0% | 1.5% |
| Categories collapsed: Strongly agree+agree, strongly disagree+disagree; very important+important. | | | | |

**Table S2:** **Percentage agreement for statements that did not achieve consensus (>80%) following Round 1 (N=32).**

|  | Agree | Neither | Disagree | Unsure |  |
| --- | --- | --- | --- | --- | --- |
| Long COVID is mainly experienced by those who had severe symptoms when they contracted COVID-19. | 10.3% | 11.7% | 71.1% | 8.1% | |
| People living with long COVID should be encouraged to participate in regular physical activities if symptoms allow. | 48.2% | 14.3% | 30.5% | 8.8% | |
| People living with long COVID can expect to make a full recovery. | 11% | 26.8% | 22.8% | 41.2% | |
| Individuals living with long COVID should receive a different form of rehabilitation than those who were hospitalised with COVID-19. | 60.3% | 16.2% | 11% | 12.5% | |
| People who have been hospitalised with COVID-19 should receive rehabilitation in hospital until they are discharged. | 46.8% | 14.8% | 19.5% | 18.8% | |
| People living with long COVID should complete a formal assessment before being encouraged to complete physical activity independently. | 77.6% | 8.8% | 9.5% | 4% | |
| Laboratory tests should play an important role in diagnosing and treating long COVID. | 64.3% | 14% | 9.9% | 11.8% | |
| People with long COVID should receive a psychological assessment as part of long COVID care. | 73.3% | 12.1% | 9.5% | 5.1% | |
| There is currently adequate support available from healthcare services for people living with long COVID. | 6.2% | 4% | 87.2% | 2.6% | |
| There are clear referral pathways to support for people living with long COVID. | 14.3% | 7.3% | 74.7% | 3.7% | |
| Healthcare professionals are aware of how to treat long COVID. | 9.9% | 9.2% | 74.7% | 6.2% | |
| Long COVID diagnosis and support should be based on clinical assessment. | 76.9% | 11% | 5.5% | 6.6% | |
| Respiratory rehabilitation for people with long COVID should be similar to that of pneumonia patients. | 14.7% | 18.7% | 23.1% | 43.6% | |
| Respiratory rehabilitation for people with long COVID should be similar to that of chronic obstructive pulmonary disease. | 13.5% | 19.4% | 24.2% | 42.9% | |
| Long COVID rehabilitation should be based on research of pre-existing conditions with similar symptoms (e.g., chronic fatigue syndrome and pneumonia). | 55.3% | 12.5% | 14.6% | 17.6% | |
| Long COVID rehabilitation plans should be similar for each patient. | 4.8% | 10.7% | 78% | 6.6% | |
| Those undergoing long COVID rehabilitation should be closely monitored for safety. | 79.5% | 12.1% | 4.1% | 4.4% | |
| How important is it for long COVID rehabilitation to focus on the following… | **Very Important** | **Moderately/ Slightly Important** | **Neither** | **Not Important** | **Unsure** |
| Anxiety | 66.8% | 24.7% | 3.3% | 2.6% | 2.6% |
| Depression | 66.9% | 25.6% | 3% | 2.2% | 2.2% |
| Exercise capacity | 75.3% | 18.1% | 1.8% | 3% | 1.8% |
| Psychological distress | 77.7% | 17.1% | 1.9% | 1.5% | 1.9% |
| Emotional support | 79.4% | 16.9% | 1.5% | 1.1% | 1.1% |
| Other | 54.9% | 15.9% | 4.4% | 0% | 24.7% |
| How important is it for long COVID rehabilitation to include the following… |  |  |  |  |  |
| Light to moderate physical activity (e.g., anything that raises the heart rate but still allows you to have a conversation at the same time) | 48.5% | 19.3% | 5.6% | 14.4% | 12.2% |
| Activity focusing on muscle use (e.g., Yoga Pilates, Gardening) | 58.6% | 23.1% | 5.2% | 6.7% | 6.3% |
| Increased cardiorespiratory demand as a result of daily activities (e.g., walking up and downstairs getting dressed and self-hygiene). | 69.2% | 13.4% | 4.5% | 4.5% | 8.6% |
| Flexibility and stretching exercises | 58% | 29.2% | 2.2% | 4.9% | 5.6% |
| Graded Exercise Therapy (GET) | 27.8% | 14.9% | 8.6% | 34.9% | 13.8% |
| Relaxation techniques (e.g., deep breathing, meditation, yoga, mindfulness) | 73.9% | 17.3% | 2.2% | 2.2% | 4.4% |
| Nutritional support | 75.3% | 18.9% | 0.7% | 1.5% | 3.7% |
| In person / face to face interventions | 68.80% | 16.50% | 4% | 4% | 6.60% |
| Interventions that can be completed virtually / at home. | 74.70% | 13.20% | 3.3% | 2.2% | 6.60% |
| Categories collapsed: Strongly agree+agree, strongly disagree+disagree; very important+important. | | | | | |

**Table S3: Percentage agreement for statements that achieved consensus (>80%) following Round 2 (N=17).**

|  | Agree | Neither | Disagree |  |
| --- | --- | --- | --- | --- |
| It is unknown whether individuals living with long COVID will make a full recovery. | 92.5% | 3.2% | 4.3% |  |
| Long COVID support and rehabilitation should be individualised to the patient’s needs. | 99.5% | 0.5% | 0 |  |
| Patients in hospital with COVID-19 should receive tailored rehabilitation and support before being discharged. | 90.3% | 8.1% | 1.6% |  |
| Individuals experiencing symptoms consistent with ME/CFS and PEM should be carefully supported before participating in physical activity. | 95.7% | 3.8% | 1% |  |
| Laboratory tests and functional screening assessments should be considered when diagnosing and treating long COVID. | 94.1% | 4.8% | 1.1% |  |
| There is inadequate and inconsistent support amongst all healthcare services for individuals living with long COVID. | 93% | 3.8% | 3.3% |  |
| There is a lack of clear referral pathways to support people living with long COVID throughout all healthcare settings. | 86.6% | 8.6% | 4.8% |  |
| Screening and detailed clinical assessment should be an important part of the diagnosis and support long COVID receive. | 97.3% | 2.7% | 0 |  |
| Long COVID rehabilitation and support should consider research of pre-existing conditions with similar symptoms e.g., myalgic encephalomyelitis / chronic fatigue syndrome. | 91.4% | 5.4% | 3.2% |  |
| Long COVID rehabilitation and support mechanisms should be dependent on each individuals’ symptoms. | 97.4% | 2.2% | 0.5% |  |
| Those completing long COVID rehabilitation and support interventions should have regular communication and monitoring with care providers. | 100% | 0 | 0 |  |
| **How important is it for long COVID rehabilitation and support to consider the following:** | **Very Important / Important** | **Moderately Important / Slightly Important** | **Not at all Important** | **Unsure** |
| The mental impact of living with long COVID | 92% | 7% | 1.1% | 0 |
| Tolerance to physical activity and physical activity | 98.4% | 1.6% | 0 | 0 |
| Emotional distress | 87.1% | 10.8% | 1.6% | 0.5% |
| Emotional wellbeing | 87.6% | 10.2% | 1.6% | 0.5% |
| **How important is it for long COVID support mechanisms to include:** |  |  |  |  |
| Relaxation techniques and breathing techniques (e.g., meditation, mindfulness) | 81.2% | 12.4% | 1.6% | 4.8% |
| Patient preference on how they attend their interventions and support, and what is most suitable for them at the time | 95.7% | 2.2% | 0 | 2.2% |
| Categories collapsed: Strongly agree+agree, strongly disagree+disagree; very important+important. | | | | |

**Table S4: Percentage agreement for statements that did not achieve consensus (<80%) following Round 2 (N=15).**

|  | **Agree** | **Neither** | **Disagree** |  |
| --- | --- | --- | --- | --- |
| The severity of COVID-19 symptoms at the acute phase (first 2 weeks of infection) is not an indicator of whether someone is at risk of developing long COVID. | 78.5% | 7% | 14.5% |  |
| If their symptoms allow and it is deemed safe to do so, people living with long COVID should participate in their regular physical activities. | 60.7% | 17.2% | 22% |  |
| People with long COVID should receive a psychological assessment as part of integrated long COVID support. | 69.9% | 20.4% | 9.6% |  |
| Appropriate treatment and support pathways are unknown to health care professionals. | 79.5% | 14% | 7.5% |  |
| Respiratory rehabilitation and support mechanisms for people with long COVID should consider findings from pneumonia research. | 60.8% | 32.3% | 7% |  |
| Respiratory rehabilitation and support mechanisms for people with long COVID should consider findings from chronic obstructive pulmonary disease. | 55.3% | 36% | 8.6% |  |
| How important is it for long COVID support mechanisms to include: | **Very Important / Important (%)** | **Moderately Important / Slightly Important (%)** | **Not at all Important (%)** | **Unsure (%)** |
| Low level physical activities (e.g., anything that raises the heart rate but still allows you to have a conversation at the same time) | 59.1% | 14.5% | 13.4% | 12.9% |
| Muscle strengthening activities (e.g., yoga, Pilates, gardening) | 66.1% | 21.5% | 4.8% | 7% |
| Daily activities that increase heart rate and breathing rate (e.g., walking up and down stairs, getting dressed, self-hygiene) | 65% | 12.9% | 12.9% | 9.1% |
| Flexibility and stretching exercises | 66.7% | 24.2% | 3.8% | 5.4% |
| Nutritional / Dietary knowledge and guidance | 79.5% | 15% | 1.6% | 3.8% |
| In person / face to face interventions | 59.7% | 30.6% | 4.3% | 5.4% |
| Interventions that can be completed virtually at home | 73.6% | 20.4% | 2.2% | 3.8% |
| Hybrid model of both face to face and virtual interventions | 78% | 14% | 2.2% | 5.9% |
| How important is it for Graded Exercise Therapy (GET) to be avoided by people with long COVID experiencing post exertional malaise, chronic fatigue, and post-exertional symptom exacerbation? | 67.7% | 14.5% | 8.6% | 9.1% |
| Categories collapsed: Strongly agree+agree, strongly disagree+disagree; very important+important. | | | | |

**Table S5: Percentage agreement for statements that achieved consensus (>80%) following Round 3 (N=5).**

|  | **Agree** | **Neither** | **Disagree (%)** |  |
| --- | --- | --- | --- | --- |
| Long COVID cannot be predicted by the severity of symptoms during the acute phase (first 2 weeks) of COVID-19 infection. | 88.4% | 7.2% | 4.3% |  |
| Long COVID services should offer psychological well-being support for those who require it. | 93.5% | 5.8% | 0.7% |  |
| There is a lack of understanding from healthcare professionals on how to support people with long COVID. | 91.3% | 3.6% | 5.1% |  |
| How important is it for long COVID rehabilitation and support mechanisms to include the following: | **Very Important / Important** | **Moderately Important / Slightly Important** | **Not at all important** | **Unsure** |
| Advice on modifying/adapting daily activities such as using aids to all greater functional ability. | 91.2% | 5.9% | 0.7% | 2.2% |
| A model that contains face to face and virtual sessions. | 80.3% | 13.2% | 3.6% | 2.9% |
| Categories collapsed: Strongly agree+agree, strongly disagree+disagree; very important+important. | | | | |

**Table S6: Percentage agreement for statements that did not achieve consensus (<80%) following Round 3 (N=10).**

|  | **Agree** | **Neither** | **Disagree** |  |
| --- | --- | --- | --- | --- |
| If regular physical activities do not provoke symptoms or post exertional symptom exacerbation, then people with long COVID can participant in their regular physical activities. | 68.9% | 18.8% | 12.3% |  |
| Those designing support mechanisms for Long COVID can learn lessons from other acute respiratory infections (e.g., pneumonia). | 57.3% | 28.3% | 14.5% |  |
| Those designing support mechanisms for Long COVID can learn lessons from other chronic respiratory diseases (e.g., asthma and COPD). | 60.2% | 26.8% | 13% |  |
| Graded Exercise Therapy should not be part of Long COVID rehabilitation and support services. | 70.3% | 13% | 16.7% |  |
| How important is it for long COVID rehabilitation and support mechanisms to include the following: | **Very Important / Important** | **Moderately Important / Slightly** | **Not at all Important** | **Unsure** |
| Low level physical activities (e.g., walking) that results in moderate increased in heart rate. | 52.9% | 18.1% | 18.8% | 10.1% |
| Activities incorporating muscle use. | 58.9% | 25% | 8.1% | 8.1% |
| Support to increase flexibility and functional movement proficiency. | 67.7% | 22,1% | 5.9% | 4.4% |
| Advice on nutrition and diet to support recovery. | 74.3% | 19.1% | 4.4% | 2.2% |
| Interventions should be delivered face to face and make use of specialist facilities and personnel. | 53.7% | 34.6% | 6.6% | 5.1% |
| Interventions that can be completed remotely and away from clinical settings. | 64.9% | 26.9% | 3.7% | 4.5% |
| Categories collapsed: Strongly agree+agree, strongly disagree+disagree; very important+important. | | | | |
